# Supplementary material for: Kinetic competition during the transcription cycle results in stochastic RNA processing
Source: eLife. 2014 Oct 1;3:e03939. doi: 10.7554/eLife.03939 (PMC4210818; doi:10.7554/eLife.03939)
Supplement: Supplementary file 1. — Mathematical analysis of the correlation functions. DOI: http://dx.doi.org/10.7554/eLife.03939.028 [file elife03939s001.pdf]

Mathematical Analysis of the Correlation Functions

Supplementary Information for: Coulon, Ferguson *et al.* (2014). Kinetic competition during the transcription cycle results in stochastic RNA processing. *eLife*, 3:e03939.

Contents

1 Single-transcript kinetics dominates at short delays **1**
2 Geometry of the transcription/splicing correlation functions **2**
Appendices **9**

1 Single-transcript kinetics dominates at short delays

For each time trace obtained from TS tracking, autocorrelation and crosscorrelation functions are defined as

$$G_{ab}(\tau) = \frac{\langle \delta a(t)\ \delta b(t+\tau) \rangle}{\langle a \rangle \langle b \rangle} \tag{1}$$

$$\text{with } \delta a(t) = a(t) - \langle a \rangle \text{ and } \delta b(t) = b(t) - \langle b \rangle \tag{2}$$

where $\langle \cdot \rangle$ denotes time average (i.e. $\langle f(t) \rangle = \lim_{T\to\infty} \frac{1}{2T} \int_{-T}^{T} f(t)dt$) and where $a(t)$ and $b(t)$ can be any combination of the red and green time traces $r(t)$ and $g(t)$ to yield all 4 correlation functions. Let $a_0(t)$ and $b_0(t)$ be the fluorescence signals from a single transcript. We first consider all the transcripts to follow the same kinetics. Let $G^0_{ab}(\tau)$ be the correlation function between $a_0(t)$ and $b_0(t)$, i.e. for a single transcription event. In this context, where the signals to be correlated are non-null only over a finite time-domain (i.e. square-integrable), the correlation function is defined as

$$G^0_{ab}(\tau) = \frac{\int_{\mathbb{R}} a_0(t) b_0(t+\tau)dt}{\int_{\mathbb{R}} a_0(t)dt\ \int_{\mathbb{R}} b_0(t)dt} \tag{3}$$

i.e. temporal averages $\langle \cdot \rangle$ in eq. (1) are replaced here by temporal sums $\int_{\mathbb{R}} \cdot\, dt$ (see Appendix 1). If initiation is considered a homogenous Poisson process (i.e. at each instant, the gene has a constant probability to fire, given by the initiation rate κ) then [1, 2] (Appendix 1)

$$G_{ab}(\tau) = \frac{G^0_{ab}(\tau)}{\kappa} \tag{4}$$

This simply reflects the fact that the fluorescence emitted by one transcript in $a(t)$ and $b(t)$ is statistically uncorrelated with that of all the other transcripts. Intuitively, since transcripts

can initiate any time (with uniform statistics) and independently to each other, their pairwise correlation of occurrence do not show any time dependence, resulting in a flat null baseline (Figure 3—figure supplement 1 and Video 4).

In the case where initiation events do not arrive with a constant rate (e.g. reflecting a bursting process or cell cycle effects), this adds an extra term to eq. (4) that is the autocorrelation of the time-dependent initiation rate $\kappa(t)$, convolved with $G^0_{ab}(\tau)$. Unless the processes causing these fluctuations are very rapid and occur at a similar timescale as the transcription/splicing process (i.e. faster than ∼300 sec), the extra term in eq. (4) only consists in a slow decay that can be approximated as a constant baseline offset in our region of interest (i.e. typically $0 \leq \tau <\sim 300$ sec). This is a reasonable assumption in our case since (i) the slow decaying component in the correlation functions is often well separated from the fast transcription/splicing kinetics (Figure 2—figure supplement 1B-F), (ii) bursting and cell cycle fluctuations (which can be observed in long acquisitions with a slow frame rate) occur at a significantly slower timescale than the duration of our time traces, and (iii) only the parts of the time traces where a non-null signal is visible and trackable are retained for analysis, effectively making the measurement to describe mostly the intra-burst kinetics rather than the bursting kinetics itself. Hence, we used eq. (4) in which the correlation functions of the whole fluorescence time traces simply boils down to the correlation functions for individual transcripts, scaled by the initiation rate κ.

2 Geometry of the transcription/splicing correlation functions

In this section, we describe how the shape of the correlation functions is mathematically linked to the timing of the transcription and splicing process. We do not assume mechanisms such as the interdependence between transcription and splicing or the kinetics of elongation, pausing and termination/3' end processing. Such assumptions about the underlying mechanisms are made in 'Mechanistic models for RNA synthesis and processing' in the 'Materials and methods' section of the main article. Here, we simply describe how the geometry of the fluorescence time profiles translate into the geometry of the correlation functions.

Simplified construct

The following aims at giving an intuition about the information content of the correlation functions and how their shape relates to the underlying timing of transcription and splicing. We make here two approximations that we will drop in the subsequent sections. Namely, we assume that (i) the kinetics of all transcripts is identical, and (ii) the MS2 and PP7 cassettes are small enough to consider that the fluorescence profiles can be described using step functions (Figure 2—figure supplement 2A).

The time course of the fluorescence emitted by a single transcript displays 4 specific events: the rise and fall of either the red or the green signal. We show here that the correlation functions reflect the time delay between any two of these 4 time points.

We consider the construct depicted on Figure 2—figure supplement 2A. We note t_r, t_g and t_{rg} respectively the time delays between rise and fall in the red signal, rise and fall in the

green signal, and rises in red and green. Because the red signal cannot fall after the green, we have $t_r \leq t_{rg} + t_g$.

The fluorescence time profiles in both colors for a single transcript can be written

$$r_0(t) = -\overline{H}(t) + \overline{H}(t - t_r) \tag{5}$$

$$g_0(t) = -\overline{H}(t - t_{rg}) + \overline{H}(t - t_{rg} - t_g) \tag{6}$$

where $t = 0$ is considered to be the rise in the red signal, $H(\cdot)$ is the Heaviside function (with the convention $H(0) = \frac{1}{2}$) and the bar denotes time reversion, i.e. the $\overline{H}(t) = H(-t)$. For convenience in the following, everything is written in terms of $\overline{H}(\cdot)$ instead of $H(\cdot)$. Time reversed versions of these functions are

$$\overline{r_0}(t) = -\overline{H}(t + t_r) + \overline{H}(t) \tag{7}$$

$$\overline{g_0}(t) = -\overline{H}(t + t_{rg} + t_g) + \overline{H}(t + t_{rg}) \tag{8}$$

The general formulation of a correlation function using the convolution product $*$ is

$$G^0_{ab}(t) = \frac{(\overline{a_0} * b_0)(t)}{\int_{\mathbb{R}} a_0(t)dt \int_{\mathbb{R}} b_0(t)dt} \tag{9}$$

Substituting eqs. (5) to (8) into eqs. (9) and (4) and dropping all the terms that are null for $t \geq 0$, we find that

$$G_{rr}(t) = \frac{\overline{H_2}(t - t_r)}{\kappa\, t_r^2} \tag{10}$$

$$G_{gg}(t) = \frac{\overline{H_2}(t - t_g)}{\kappa\, t_g^2} \tag{11}$$

$$G_{rg}(t) = \frac{\overline{H_2}(t - t_{rg} + t_r) - \overline{H_2}(t - t_{rg} + t_r - t_g) - \overline{H_2}(t - t_{rg}) + \overline{H_2}(t - t_{rg} - t_g)}{\kappa\, t_r t_g} \tag{12}$$

$$G_{gr}(t) = \frac{\overline{H_2}(t + t_{rg} - t_r)}{\kappa\, t_r t_g} \tag{13}$$

We used the notation $\overline{H_2}(t) = \overline{H}(t) * \overline{H}(t)$, i.e. a time-reversed ramp function and the property that $\overline{H}(t - t_1) * \overline{H}(t - t_2) = \overline{H_2}(t - t_1 - t_2)$.

We should note that, even though these equations are defined for $t \geq 0$, eq. (12) is actually valid on $\mathbb{R}$ since no term was found null in its calculation and hence omitted. Indeed, the first term of eq. (12) corresponds to the time-reversed version of eq. (13) and all three other terms are null on $t \leq 0$ by construction. Hence, eq. (12) is the two-sided expression of $G_{rg}(t)$.

These correlation functions (Figure 2—figure supplement 2A) are piece-wise linear with angles revealing specific time delays in the underlying process (i.e. each $\overline{H_2}(\cdot)$ term is an angle). Namely, these are all the delays from the 6 possible pairs obtained by combining the rising and falling edges of the red and green signals. The 2 pairs between edges of the same color are in the autocorrelations and the 4 pairs mixing both colors are in the crosscorrelation.

A few geometrical conclusions are worth pointing out:

- In this context where transcripts are identical (Figure 2—figure supplement 2C), we observe:
  - a null $G_{rg}(0)$ if splicing is co-transcriptional and occurs before the MS2 cassette is transcribed ($t_r < t_{rg}$).
  - a non-null $G_{rg}(0)$ with a positive slope at 0^+ if splicing occurs between transcription of the MS2 cassette and release of the transcript ($t_{rg} < t_r < t_{rg} + t_g$). In that case, it equals the slope at 0^- (obtained from $\overline{G_{gr}}(t)$).
  - a non-null $G_{rg}(0)$ with a null slope at 0^+ if splicing is post-release ($t_r = t_{rg} + t_g$).

  None of these scenarios are observed in our experimental data (Fig. 2B, inset) since $G_{rg}(0)$ is always non-null and the slope of $G_{rg}(0^+)$ is strictly positive but not aligned with that of $G_{rg}(0^-)$.
- The delay where the red-to-green crosscorrelation $G_{rg}(t)$ starts decreasing measures the time to elongate between both cassettes, independently of any upstream (e.g. initiation, proximal pausing) and downstream effects (e.g. termination). From Fig. 2B (inset) and given the distance between the centers of both cassettes, we estimate graphically that the elongation rate is around $\sim$2.5kb/min.
- The duration of the green signal (from rise to fall) appears in the decay of both autocorrelation $G_{gg}(t)$ and the last segment of crosscorrelation $G_{rg}(t)$. Conversely, the duration of the red signal appears in both autocorrelation $G_{rr}(t)$ and the rising part of the two-sided crosscorrelation made of $\overline{G_{gr}}(t)$ and $G_{rg}(t)$. In addition, the fall of the green signal (release of the RNA) imposes the red signal to fall with it (the RNA is unspliced) if it has not already. As a consequence, if some process delays the fall in green (e.g. a slow termination kinetics) without making it substantially longer than the splicing time, then the tails of all four correlation curves are expected to get longer and show similar decay. This is what is observed in our experimental data (Fig. 2B).

Ramped construct

Here, we drop the simplification that the cassettes are short enough so that the rises of fluorescence can be approximated by step functions. Instead, we consider ramps that span the entire width of the cassettes, describing the progressive increases of fluorescence as the polymerase progresses through these regions (Figure 2—figure supplement 2B). We show that the sharp angles in the correlation functions described previously section turn into smooth angles since they originate from the convolution between fluorescence transitions that are no longer instantaneous.

We note t_{r1} and t_{g1} the time to elongate through the red and the green cassettes, t_{r2} and t_{g2} the time between the end of the ramps and the fall of the signal, and t_{rg} the time between the beginning of both ramps. By construction, we have $t_{r1} < t_{rg}$, and because the red signal cannot fall after the green, we have $t_{r1} + t_{r2} \leq t_{rg} + t_{g1} + t_{g2}$.

The fluorescence time profiles for a single transcript and their time-reversed versions are

$$r_0(t) = \frac{\overline{H_2}(t) - \overline{H_2}(t - t_{r1})}{t_{r1}} + \overline{H}(t - t_{r1} - t_{r2}) \tag{14}$$

$$g_0(t) = \frac{\overline{H_2}(t - t_{rg}) - \overline{H_2}(t - t_{rg} - t_{g1})}{t_{g1}} + \overline{H}(t - t_{rg} - t_{g1} - t_{g2}) \tag{15}$$

$$\overline{r_0}(t) = -\overline{H}(t + t_{r1} + t_{r2}) + \frac{-\overline{H_2}(t + t_{r1}) + \overline{H_2}(t)}{t_{r1}} \tag{16}$$

$$\overline{g_0}(t) = -\overline{H}(t + t_{rg} + t_{g1} + t_{g2}) + \frac{-\overline{H_2}(t + t_{rg} + t_{g1}) + \overline{H_2}(t + t_{rg})}{t_{g1}} \tag{17}$$

Substituting again eqs. (14) to (17) into eqs. (9) and (4) and dropping all the terms that are null for $t \geq 0$, we now find that

$$G_{rr}(t) = \frac{\kappa}{\langle r\rangle^2}\left(\frac{-\overline{H_4}(t - t_{r1})}{t_{r1}^2} + \frac{-\overline{H_3}(t - t_{r2}) + \overline{H_3}(t - t_{r1} - t_{r2})}{t_{r1}}\right) \tag{18}$$

$$G_{gg}(t) = \frac{\kappa}{\langle g\rangle^2}\left(\frac{-\overline{H_4}(t - t_{g1})}{t_{g1}^2} + \frac{-\overline{H_3}(t - t_{g2}) + \overline{H_3}(t - t_{g1} - t_{g2})}{t_{g1}}\right) \tag{19}$$

$$\begin{aligned} G_{rg}(t) = \frac{\kappa}{\langle r\rangle\langle g\rangle}\Bigg(& \frac{-\overline{H_3}(t + t_{r1} + t_{r2} - t_{rg}) + \overline{H_3}(t + t_{r1} + t_{r2} - t_{rg} - t_{g1})}{t_{g1}} \\ & - \overline{H_2}(t + t_{r1} + t_{r2} - t_{rg} - t_{g1} - t_{g2}) \\ & - \frac{\overline{H_4}(t + t_{r1} - t_{rg}) - \overline{H_4}(t + t_{r1} - t_{rg} - t_{g1}) - \overline{H_4}(t - t_{rg}) + \overline{H_4}(t - t_{rg} - t_{g1})}{t_{g1}\, t_{r1}} \\ & + \frac{-\overline{H_3}(t + t_{r1} - t_{rg} - t_{g1} - t_{g2}) + \overline{H_3}(t - t_{rg} - t_{g1} - t_{g2})}{t_{r1}}\Bigg) \end{aligned} \tag{20}$$

$$G_{gr}(t) = \frac{\kappa}{\langle r\rangle\langle g\rangle}\left(\frac{-\overline{H_3}(t + t_{rg} + t_{g1} - t_{r1} - t_{r2}) + \overline{H_3}(t + t_{rg} - t_{r1} - t_{r2})}{t_{g1}}\right) \tag{21}$$

where $\overline{H_n}(t) = \overline{H_{n-1}}(t) * \overline{H}(t) = (-t)^{n-1}/(n-1)!$ if $t \leq 0$ and 0 elsewhere, and with $\langle r\rangle = \kappa\,(t_{r1}/2 + t_{r2})$ and $\langle g\rangle = \kappa\,(t_{g1}/2 + t_{g2})$.

Although eqs. (18) to (21) are defined for $t \geq 0$, eq. (20) is actually valid on $\mathbb{R}$ since no term was found null in its calculation and hence omitted. Indeed, the first line of eq. (20) is to the time-reversed version of eq. (21), making eq. (20) the two-sided expression of $G_{rg}(t)$.

It is instructive to compare these new correlation functions with those obtained in the previous section with simplified fluorescence profiles (Figure 2—figure supplement 2A and B). We realize that the sharp angles previously identifying the well-defined delays between rapid fluorescence transitions (terms in $\overline{H_2}(\cdot)$ in eqs. (10) to (13) originating from the convolution of pairs of step functions) are now replaced[1], for most of them, by smooth angles reflecting

[1] A term of the form $\frac{-\overline{H_3}(t-a) + \overline{H_3}(t-a-b)}{b}$ yields a curve that is rounded on $a \leq t \leq b$ but straight

the less-defined delays between a ramp and a step (terms in $\overline{H_3}(\cdot)$ in eqs. (18) to (21)) or between two ramps (terms in $\overline{H_4}(\cdot)$). Only the angle reflecting the well-defined delay between the rapid falls of both fluorescence signals remains sharp, i.e. the one revealing the occurrence of splicing relatively to transcript release.

Like with the simplified construct, the slopes of $G_{rg}(t)$ around $t = 0$ also indicate the post- or co-release nature of the splicing:

First, only the two first lines of eq. (20) can contribute to the slopes of $G_{rg}(t)$ around 0. Indeed, by construction, the red ramp always finishes before the green ramp starts ($t_{r1} < t_{rg}$) and before the green signal falls. Thus, the two rightmost angles in $G_{rg}(t)$ (two last lines of eq. (20)) have no influence on the slopes at 0, i.e. they both equal a term in $\overline{H_2}(\cdot)$ with opposite sign, yielding a null contribution to the slope.

Hence, for t in the neighborhood of 0, we have

$$\frac{\langle r\rangle\langle g\rangle}{\kappa}\,\frac{d}{dt}G_{rg}(t) = \frac{\overline{H_2}(t+t_{r1}+t_{r2}-t_{rg}) - \overline{H_2}(t+t_{r1}+t_{r2}-t_{rg}-t_{g1})}{t_{g1}} + \overline{H}(t+t_{r1}+t_{r2}-t_{rg}-t_{g1}-t_{g2}) \tag{22}$$

$$= g_0(t+t_{r1}+t_{r2}) \tag{23}$$

i.e., the slopes of $G_{rg}(t)$ at 0^- and 0^+ are proportional to the value of the green signal respectively right before and right after the fall of the red signal. Hence, if splicing is post-release (i.e. both red and green signals fall together), there is a break of slope at 0 since $\frac{\langle r\rangle\langle g\rangle}{\kappa}\,\frac{d}{dt}G_{rg}(t)$ equals 1 at 0^- and is null at 0^+. Otherwise, splicing occurs before release and both slopes are equal and positive.

Another way of reaching this conclusion is by noting that the sharp angle due to the term in $\overline{H_2}(\cdot)$ in eq. (20) is at $t = 0$ if splicing is post-release and at $t > 0$ otherwise, making the two slopes to differ only in the former case.

Stochastic transcript kinetics

The explicit form of eq. (4) we used so far is

$$G_{ab}(\tau) = \frac{1}{\kappa}\,\frac{\int_{\mathbb{R}} a_0(t)b_0(t+\tau)dt}{\int_{\mathbb{R}} a_0(t)dt\,\int_{\mathbb{R}} b_0(t)dt} \tag{24}$$

This equation assumes that all transcripts have identical kinetics, described by signals $a_0(t)$ and $b_0(t)$. When each transcript is different, this equation generalizes to (see Appendix 1)

$$G_{ab}(\tau) = \frac{\left\langle \int_{\mathbb{R}} a_i(t)b_i(t+\tau)dt\right\rangle_i}{\kappa\left\langle \int_{\mathbb{R}} a_i(t)dt\right\rangle_i\left\langle \int_{\mathbb{R}} b_i(t)dt\right\rangle_i} \tag{25}$$

---

and equal to $\overline{H_2}(t-a-b/2)$ anywhere else.

Similarly, a term of the form $\frac{\overline{H_4}(t-a) - \overline{H_4}(t-a-b) - \overline{H_4}(t-a-c) + \overline{H_4}(t-a-c-b)}{b\,c}$ yields a curve that is rounded on $a \le t \le b+c$ but straight and equal to $\overline{H_2}(t-a-b/2-c/2)$ anywhere else. Hence, each line in eqs. (18) to (21) corresponds to a rounded version of each terms in eqs. (10) to (13).

where $a_i(t)$ and $b_i(t)$ are the contributions of transcript i to the total fluorescence signals $a(t)$ and $b(t)$, and $\langle\cdot\rangle_i$ denotes the average over all transcripts. In other words, up to some normalization constants, the correlation function resulting from a heterogeneous population of transcripts is simply the average of the correlation functions of each transcript.

To get rid of the normalization constants, one can formulate this equation in terms of covariance (i.e. unnormalized central moments $M_{ab}(\tau) = \langle \delta a(t)\, \delta b(t+\tau) \rangle\rangle$ as follows

$$M_{ab}(\tau) = \langle a \rangle \langle b \rangle G_{ab}(\tau) = \kappa \left\langle \int_{\mathbb{R}} a_i(t) b_i(t+\tau) dt \right\rangle_i \tag{26}$$

Fraction of pre-release splicing: Change of slope at the origin

Due to the stochastic kinetics of transcription and splicing, only a fraction of transcripts may be spliced before release while the rest is spliced after release. In that case, the slopes of $G_{rg}(t)$ around 0 will reflect this heterogeneity (Figure 2—figure supplement 2D):

The denominator of eq. (25) is constant with respect to τ and equals[2] $\frac{\langle r \rangle \langle g \rangle}{\kappa}$. Hence, eq. (23) simply generalized into

$$\frac{\langle r \rangle \langle g \rangle}{\kappa} \frac{d}{dt} G_{rg}(t) = \left\langle g_i(t - t_i^{\text{red}\downarrow}) \right\rangle_i \tag{27}$$

indicating that the slopes at 0^- and 0^+ are proportional to the average values of the green signal respectively right before and right after the fall of the red signal (noted $t_i^{\text{red}\downarrow}$). Since, these two values differ by 1 for all transcripts spliced post-release, and by 0 for all transcripts spliced pre-release, then the difference

$$p_{\text{post}} = \frac{\langle r \rangle \langle g \rangle}{\kappa} \left(\left.\frac{dG_{rg}(t)}{dt}\right|_{0^-} - \left.\frac{dG_{rg}(t)}{dt}\right|_{0^+} \right) \tag{28}$$

measures exactly the fraction of transcripts that are spliced post-release. However, the prefactor in this expression cannot be obtained exactly from the correlation curves. The slope at 0^- provides an approximation

$$\left.\frac{dG_{rg}(t)}{dt}\right|_{0^-} \lesssim \frac{\kappa}{\langle r \rangle \langle g \rangle} \tag{29}$$

This approximation is exact only if splicing always happens after the ramp in the green signal (i.e. after elongating through the MS2 cassette), and is an underestimation otherwise.

Combining eqs. (28) and (29), the fraction of pre-release splicing can be estimated by

$$p_{\text{pre}} \gtrsim \frac{dG_{rg}(t)/dt|_{0^+}}{dG_{rg}(t)/dt|_{0^-}} \tag{30}$$

constituting an approximation that can only underestimate the actual value.

[2]Because $\langle r \rangle = \kappa \left\langle \int_{\mathbb{R}} r_i(t) dt \right\rangle_i$ and $\langle g \rangle = \kappa \left\langle \int_{\mathbb{R}} g_i(t) dt \right\rangle_i$.

Hence, the orientation of $G_{rg}(0^+)$ between horizontal and aligned with $G_{rg}(0^-)$ provides a measure of the pre-release fraction (Figure 2—figure supplement 2D).

Using our experimental correlation curves (Fig. 2B and Figure 2—figure supplement 3A), we computed the two slopes by fitting the time derivatives of the crosscorrelation functions to a constant value on either side of $\tau = 0$, using the points highlighted in fig Figure 2—figure supplement 3B. SEM of the derivatives were obtained from bootstrapping and propagated to yield SEM on the slope values. We concluded that splicing occurs pre-release for at least 13%(±5%) of the transcripts. In addition, we performed two two-sided z-tests to compute a p-value of less than 0.003 indicating that splicing is neither all pre-release nor all post-release. These tested respectively if the slope of $G_{rg}(\tau)$ is (i) different from 0 and (ii) different from the slope of $G_{gr}(\tau)$. Both p-values p_1 and p_2 were then combined into a single p-value $1 - (1 - p_1)(1 - p_2) < 0.003$ indicating that both outcome are occurring.

Figure 2—figure supplement 3C illustrate with simulated data how accurately this method is able to estimate the actual pre-release fraction. Stochastic simulations were performed with two of the models presented in the next section, using parameters obtained from fitting the experimental data (apart from the splicing time which is varied). Each point on the main panel of Figure 2—figure supplement 3C corresponds to the same amount of data as for the experimental correlation functions (i.e. 23 traces of 245 frames on average). These figures indicate that the ratio of crosscorrelation slopes is an accurate estimate of the actual pre-release fraction, even though it has relatively large errors and often slightly underestimate the actual value. The inset panel uses more simulated data, yielding more precise estimates.

Appendices

Appendix 1

The transcriptional time traces $a(t)$ and $b(t)$ can be viewed as the sums

$$a(t) = \sum_i a_i(t) \qquad b(t) = \sum_i b_i(t) \tag{31}$$

where $a_i(t)$ and $b_i(t)$ are the contribution of transcript i to the signals. From the definition of the correlation functions (1), it follows that

$$G_{ab}(\tau) = \frac{\langle \delta a(t)\ \delta b(t+\tau)\rangle}{\langle a\rangle\langle b\rangle} \quad \text{with } \delta a(t) = a(t) - \langle a\rangle \text{ and } \delta b(t) = b(t) - \langle b\rangle \tag{32}$$

$$= \frac{\langle a(t)\ b(t+\tau)\rangle}{\langle a\rangle\langle b\rangle} - 1 \tag{33}$$

$$= \frac{\left\langle \sum_i a_i(t)\ b_i(t+\tau)\right\rangle + \left\langle \sum_i \left[a_i(t)\sum_{j\neq i} b_j(t+\tau)\right]\right\rangle}{\left\langle \sum_i a_i(t)\right\rangle\left\langle \sum_j b_j(t)\right\rangle} - 1 \tag{34}$$

Each of the above terms corresponding to a temporal average of a sum over all the transcripts (e.g. $\left\langle \sum_i a_i(t)\right\rangle$) can be turned[3] into an average over all the transcripts of a temporal sum (e.g. $\kappa\left\langle \int_{\mathbb{R}} a_i(t)dt\right\rangle_i$), yielding

$$G_{ab}(\tau) = \frac{\kappa\left\langle \int_{\mathbb{R}} a_i(t)b_i(t+\tau)dt\right\rangle_i + \kappa\left\langle \int_{\mathbb{R}} \left[a_i(t)\sum_{j\neq i} b_j(t+\tau)\right]dt\right\rangle_i}{\kappa^2\left\langle \int_{\mathbb{R}} a_i(t)dt\right\rangle_i\left\langle \int_{\mathbb{R}} b_j(t)dt\right\rangle_j} - 1 \tag{35}$$

If transcription initiation has homogeneous Poisson statistics, the occurrence of transcripts i and j is uncorrelated. In that context, $a_i(t)$ and $\sum_{j\neq i} b_j(t+\tau)$ are independent stochastic signals, so that the latter can be replaced in the equation above by its temporal mean $\left\langle \sum_{j\neq i} b_j(t)\right\rangle$ which simplifies into $\kappa\left\langle \int_{\mathbb{R}} b_j(t)dt\right\rangle_j$ in the limit of many transcripts.

This yields

$$G_{ab}(\tau) = \frac{\kappa\left\langle \int_{\mathbb{R}} a_i(t)b_i(t+\tau)dt\right\rangle_i + \kappa^2\left\langle \int_{\mathbb{R}} a_i(t)dt\right\rangle_i\left\langle \int_{\mathbb{R}} b_j(t)dt\right\rangle_j}{\kappa^2\left\langle \int_{\mathbb{R}} a_i(t)dt\right\rangle_i\left\langle \int_{\mathbb{R}} b_j(t)dt\right\rangle_j} - 1 \tag{36}$$

$$= \frac{\left\langle \int_{\mathbb{R}} a_i(t)b_i(t+\tau)dt\right\rangle_i}{\kappa\left\langle \int_{\mathbb{R}} a_i(t)dt\right\rangle_i\left\langle \int_{\mathbb{R}} b_j(t)dt\right\rangle_j} \tag{37}$$

If all transcripts are identical, eq. (37) simplifies into

$$G_{ab}(\tau) = \frac{\int_{\mathbb{R}} a_0(t)b_0(t+\tau)dt}{\kappa\int_{\mathbb{R}} a_0(t)dt\int_{\mathbb{R}} b_0(t)dt} \tag{38}$$

$$\stackrel{\text{def}}{=} \frac{G^0_{ab}(\tau)}{\kappa} \tag{39}$$

[3] More explicitly,

$$\left\langle \sum_i a_i(t)\right\rangle = \lim_{T\to\infty} \frac{1}{2T}\int_{-T}^{T} \sum_i a_i(t)dt$$

$$= \lim_{T\to\infty} \frac{\kappa\,2T}{2T}\left\langle \int_{-T}^{T} a_i(t)dt\right\rangle_i \quad \text{where } \langle\cdot\rangle_i = \frac{\sum_i \cdot}{\kappa\,2T} \text{ is the average over all the transcripts.}$$

$$= \kappa\left\langle \int_{\mathbb{R}} a_i(t)dt\right\rangle_i$$

Note that eq. (37) expresses the correlation between *square-integrable* signals $a_i(t)$ and $b_i(t)$ (i.e. although defined on $\mathbb{R}$, their non-null part is of finite duration), whence the use of temporal *sums*. On the contrary, eq. (32) uses *non-square-integrable* signals $a(t)$ and $b(t)$ (i.e. infinite signals), whence the use of temporal *averages* and *mean-subtracted* signals.

Appendix 2

Appendix 2A – Scheme I

We show that, using the distributions of scheme I (Figure 2—figure supplement 4), the correlation functions are

$$G_{rr}(t) = \frac{\kappa}{\langle r\rangle^2}\ \overline{H_2} * \left(-\overline{H_2} * \frac{A}{t^2} + \overline{H} * \left(-\frac{\delta}{h_A} + \frac{A}{t}\right) * B * D * E\right) \tag{40}$$

$$G_{gg}(t) = \frac{\kappa}{\langle g\rangle^2}\ \overline{H_2} * \left(-\overline{H_2} * \frac{D}{t^2} + \overline{H} * \left(-\frac{\delta}{h_D} + \frac{D}{t}\right) * E\right) \tag{41}$$

$$G_{rg}(t) = \frac{\kappa}{\langle r\rangle\langle g\rangle}\ \overline{H_3} * \left(-\frac{\delta}{h_A} + \frac{A}{t}\right) * B * \left(-\overline{H} * \left(-\frac{\delta}{h_D} + \frac{D}{t}\right) + D * E\right) \tag{42}$$

$$G_{gr}(t) = \frac{\kappa}{\langle r\rangle\langle g\rangle}\ \overline{H_3} * \left(-\frac{\delta}{h_D} + \frac{D}{t}\right) * E \tag{43}$$

with $\langle r\rangle = \kappa(\mu_A/2 + \mu_B + \mu_D + \mu_E)$ and $\langle g\rangle = \kappa(\mu_D/2 + \mu_E)$

μ_X and h_X represent the arithmetic and harmonic means of a distribution X.

To simplify the calculations, we first derive the expressions of the covariance functions $M_{ab}(\tau) = \langle \delta a(t)\ \delta b(t+\tau)\rangle$ before normalizing them to obtain the correlation functions $G_{ab}(\tau) = \frac{M_{ab}(\tau)}{\langle a\rangle\langle b\rangle}$. We note $M_{ab}^{\det}(\tau)$ the covariance functions derived in the deterministic context of eqs. (18) to (21). In the stochastic context of scheme I, eq. (26) rewrites

$$M_{ab}(t) = \iiiint_{\mathbb{R}^4} \left[M_{ab}^{\det}(t)\right] A(t_A)\, B(t_B)\, D(t_D)\, E(t_E)\ dt_A\, dt_B\, dt_D\, dt_E \tag{44}$$

where $t_{r1} = t_A$
$t_{r2} = t_B + t_D + t_E$
$t_{g1} = t_D$
$t_{g2} = t_E$
$t_{rg} = t_A + t_B$

For instance, the green autocovariance reads

$$M_{gg}(t) = \kappa \iiiint_{\mathbb{R}^4} \left[\frac{-\overline{H_4}(t - t_D)}{t_D^2} + \frac{-\overline{H_3}(t - t_E) + \overline{H_3}(t - t_D - t_E)}{t_D}\right] A(t_A)\, B(t_B)\, D(t_D)\, E(t_E)\ dt_A\, dt_B\, dt_D\, dt_E \tag{45}$$

Because the expression between the brackets is independent of t_A, the term $\int_{\mathbb{R}} A(t_A) dt_A$ (which equals 1) can be factorized out. The same is true for t_B, yielding

$$M_{gg}(t) = \kappa \iint_{\mathbb{R}^2} \left[\frac{-\overline{H_4}(t - t_D)}{t_D^2} + \frac{-\overline{H_3}(t - t_E) + \overline{H_3}(t - t_D - t_E)}{t_D}\right] D(t_D) E(t_E)\ dt_D dt_E \tag{46}$$

The same also holds for t_E with respect to the term in $\overline{H_4}$. Moreover, for any function $f(t)$, $\int_{\mathbb{R}} f(t - t_E)\, E(t_E) dt_E = f(t) * E(t)$ is the definition of the convolution product. Thus,

$$M_{gg}(t) = \kappa \int_{\mathbb{R}} \left[\frac{-\overline{H_4}(t - t_D)}{t_D^2} + \frac{-\overline{H_3}(t) + \overline{H_3}(t - t_D)}{t_D} * E(t)\right] D(t_D)\ dt_D \tag{47}$$

Then, similarly, $\int_{\mathbb{R}} \overline{H_4}(t-t_D)\frac{D(t_D)}{t_D^2}dt_D = \overline{H_4}(t) * \frac{D(t)}{t^2}$ and $\int_{\mathbb{R}} \overline{H_3}(t-t_D)\frac{D(t_D)}{t_D}dt_D = \overline{H_3}(t) * \frac{D(t)}{t}$ are also convolution products. And $\overline{H_3}(t)\int_{\mathbb{R}} \frac{D(t_D)}{t_D}dt_D = \frac{\overline{H_3}(t)}{h_D}$ where h_D denotes the harmonic mean of $D(t)$. Hence, we obtain

$$M_{gg}(t) = \kappa\left[-\overline{H_4}(t) * \frac{D(t)}{t^2} + \overline{H_3}(t) * \left(-\frac{\delta(t)}{h_D} + \frac{D(t)}{t}\right) * E(t)\right] \tag{48}$$

where $\delta(t)$ is the Dirac function (e.g. $\overline{H_3}(t) * \delta(t) = \overline{H_3}(t)$), used here simply to factorize the expression. Finally, we simplify the notation by omitting all the dependencies on t, yielding

$$M_{gg}(t) = \kappa\left[-\overline{H_4} * \frac{D}{t^2} + \overline{H_3} * \left(-\frac{\delta}{h_D} + \frac{D}{t}\right) * E\right] \tag{49}$$

The red autocovariance $M_{rr}(t)$ is obtained by applying the same derivation, simply replacing distribution D by A, and distribution E by $B * D * E$, yielding to eq. (40).

The red to green crosscovariance function $M_{rg}(t)$ is derived as follows:

$$\begin{aligned} M_{rg}(t) = \kappa \iiiint_{\mathbb{R}^4} \Bigg[& \frac{-\overline{H_3}(t+t_D+t_E) + \overline{H_3}(t+t_E)}{t_D} - \overline{H_2}(t) \\ & - \frac{\overline{H_4}(t-t_B) - \overline{H_4}(t-t_B-t_D) - \overline{H_4}(t-t_A-t_B) + \overline{H_4}(t-t_A-t_B-t_D)}{t_D\, t_A} \\ & + \frac{-\overline{H_3}(t-t_B-t_D-t_E) + \overline{H_3}(t-t_A-t_B-t_D-t_E)}{t_A}\Bigg] \\ & A(t_A)\, B(t_B)\, D(t_D)\, E(t_E)\, dt_A\, dt_B\, dt_D\, dt_E \end{aligned} \tag{50}$$

Of the terms between brackets, only second and third lines are non-null over $t \geq 0$. Applying similar steps as from eqs. (46) to (49), and then factorizing, we obtain eq. (42).

Finally, the green to red crosscovariance function $M_{gr}(t)$ is derived as follows:

$$M_{gr}(t) = \kappa \iint_{\mathbb{R}^2} \left[\frac{-\overline{H_3}(t-t_E) + \overline{H_3}(t-t_D-t_E)}{t_D}\right] D(t_D)\, E(t_E)\, dt_D\, dt_E \tag{51}$$

Applying again similar steps as from eqs. (46) to (49), we obtain eq. (43).

Appendix 2B – Scheme II

We show that, using the distributions of scheme II (Figure 2—figure supplement 4), the correlation functions are

$$G_{rr}(t) = \frac{\kappa}{\langle r\rangle^2}\ \overline{H_2} * \left(-\overline{H_2} * \frac{A}{t^2} + \left(-\frac{\delta}{h_A} + \frac{A}{t}\right) * B * (\overline{H} * F)(\overline{H} * C * D * E)\right) \tag{52}$$

$$G_{gg}(t) = \frac{\kappa}{\langle g\rangle^2}\ \overline{H_2} * \left(-\overline{H_2} * \frac{D}{t^2} + \overline{H} * \left(-\frac{\delta}{h_D} + \frac{D}{t}\right) * E\right) \tag{53}$$

$$G_{rg}(t) = \frac{\kappa}{\langle r\rangle\langle g\rangle}\overline{H_2} * \left(\overline{H} * \left(-\frac{\delta}{h_A} + \frac{A}{t}\right) * B - \overline{F}\right) * C * \left(-\overline{H} * \left(-\frac{\delta}{h_D} + \frac{D}{t}\right) + D * E\right) \tag{54}$$

$$G_{gr}(t) = \frac{\kappa}{\langle r\rangle\langle g\rangle}\overline{H_2} * \left((\overline{H} * E)\left(\overline{H} * F * \overline{C} * \frac{\overline{D}}{t}\right) + \left(\overline{H} * \frac{D}{t} * E\right)(\overline{H} * F * \overline{C})\right) \tag{55}$$

with $\langle r\rangle = \kappa(\mu_A/2 + \mu_B + \mu_{\lfloor F, C*D*E\rfloor})$ and $\langle g\rangle = \kappa(\mu_D/2 + \mu_E)$

$\lfloor X, Y\rfloor$ denotes the distribution of the minimum of between two numbers drawn from distributions X and Y (i.e. $\overline{H} * \lfloor X, Y\rfloor = (\overline{H} * X)(\overline{H} * Y)$).

Similarly as for scheme I, eq. (26) rewrites in the context of scheme II as follows:

$$M_{ab}(t) = \iint\cdots\int_{\mathbb{R}^6} \left[M_{ab}^{\det}(t)\right] A(t_A)\, B(t_B) \cdots F(t_F)\; dt_A\, dt_B \cdots dt_F \tag{56}$$

$$\begin{aligned} \text{where } t_{r1} &= t_A \\ t_{r2} &= t_B + \min(t_C + t_D + t_E, t_F) \\ t_{g1} &= t_D \\ t_{g2} &= t_E \\ t_{rg} &= t_A + t_B + t_C \end{aligned}$$

The derivation of the green autocorrelation is identical to the one we performed for scheme I, yielding the exact same expression for eq. (53).

The same derivation also applies for the red autocorrelation so that, as for scheme I, it can be obtained by substituting distributions in eq. (49). However, here, distribution D is replaced by A, and distribution E by the distribution of the minimum between two random numbers drawn from distributions $B * F$ and $B*C*D*E$ (reflecting that the red signal falls when either splicing occurs or when the transcript is released). The complementary cumulative distribution function (CCDF) of the minimum between two distributions is simply the product of their CCDF[4]. Then, to obtain $M_{rr}(t)$ from eq. (49), $\overline{H} * E$ is replaced by $B * (\overline{H} * F)(\overline{H} * C * D * E)$, with the convention that the regular product precedes over the convolution product. We obtain

$$M_{rr}(t) = \kappa \left[-\overline{H_4} * \frac{A}{t^2} + \overline{H_2} * \left(-\frac{\delta}{h_A} + \frac{A}{t}\right) * B * (\overline{H} * F)(\overline{H} * C * D * E)\right] \tag{57}$$

The red to green crosscovariance function is derived as follows:

$$\begin{aligned} M_{rg}(t) = \kappa \iint\cdots\int_{\mathbb{R}^6} \Bigg[& \frac{-\overline{H_3}(t + \min(t_D + t_E, t_F - t_C)) + \overline{H_3}(t + \min(t_E, t_F - t_C - t_D))}{t_D} \\ & - \overline{H_2}(t + \min(0, t_F - t_C - t_D - t_E)) \\ & - \frac{\overline{H_4}(t-t_B-t_C) - \overline{H_4}(t-t_B-t_C-t_D) - \overline{H_4}(t-t_A-t_B-t_C) + \overline{H_4}(t-t_A-t_B-t_C-t_D)}{t_D\, t_A} \\ & + \frac{-\overline{H_3}(t - t_B - t_C - t_D - t_E) + \overline{H_3}(t - t_A - t_B - t_C - t_D - t_E)}{t_A} \Bigg] \\ & A(t_A)\, B(t_B) \cdots F(t_F)\; dt_A\, dt_B \cdots dt_F \end{aligned} \tag{58}$$

Noting that, over $t \geq 0$, a term of the form $\overline{H_n}(t + \min(a, b))$ simplifies to $\overline{H_n}(t + b)$ if $a \geq 0$, and applying similar steps as from eq. (46) to (49), then factorizing, we find

$$M_{rg}(t) = \kappa\, \overline{H_2} * \left(\overline{H} * \left(-\frac{\delta}{h_A} + \frac{A}{t}\right) * B - \overline{F}\right) * C * \left(-\overline{H} * \left(-\frac{\delta}{h_D} + \frac{D}{t}\right) + D * E\right) \tag{59}$$

Finally, the green to red crosscovariance function is derived as follows:

$$M_{gr}(t) = \kappa \iiiint_{\mathbb{R}^4} \left[\frac{-\overline{H_3}(t - \min(t_E, t_F - t_C - t_D)) + \overline{H_3}(t - \min(t_D + t_E, t_F - t_C))}{t_D}\right] C(t_C)\, D(t_D)\, E(t_E)\, F(t_F)\; dt_C\, dt_D\, dt_E\, dt_F \tag{60}$$

$$= \kappa\, \overline{H_2}(t) * \iiiint_{\mathbb{R}^4} \left[\frac{-\overline{H}(t - t_E)\overline{H}(t - t_F + t_C + t_D) + \overline{H}(t - t_D - t_E)\overline{H}(t - t_F + t_C)}{t_D}\right] C(t_C)\, D(t_D)\, E(t_E)\, F(t_F)\; dt_C\, dt_D\, dt_E\, dt_F \tag{61}$$

$$= \kappa\, \overline{H_2} * \left((\overline{H} * E)\left(\overline{H} * F * \overline{C} * \frac{\overline{D}}{t}\right) + \left(\overline{H} * \frac{D}{t} * E\right)(\overline{H} * F * \overline{C})\right) \tag{62}$$

[4]The CCDF of any distribution f simply reads $\int_t^{-\infty} f(t')dt' = \int_{\mathbb{R}} \overline{H}(t - t')f(t')dt' = \overline{H} * f$.

Appendix 2C – Scheme III

We show that, using the distributions of scheme III (Figure 2—figure supplement 4), the correlation functions are

$$G_{rr}(t) = \frac{\kappa}{\langle r\rangle^2}\ \overline{H_2} * \left(-\overline{H_2} * \frac{A}{t^2} + \overline{H} * \left(-\frac{\delta}{h_A} + \frac{A}{t}\right) * B * F\right) \tag{63}$$

$$G_{gg}(t) = \frac{\kappa}{\langle g\rangle^2}\ \overline{H_2} * \left(-\overline{H_2} * \frac{D}{t^2} + \overline{H} * \left(-\frac{\delta}{h_D} + \frac{D}{t}\right) * E\right) \tag{64}$$

$$G_{rg}(t) = \frac{\kappa}{\langle r\rangle\langle g\rangle}\ \overline{H_2} * \left(\overline{H} * \left(-\frac{\delta}{h_A} + \frac{A}{t}\right) * B * F - \delta\right) * C * \left(-\overline{H} * \left(-\frac{\delta}{h_D} + \frac{D}{t}\right) + D * E\right) \tag{65}$$

$$G_{gr}(t) = 0 \tag{66}$$

with $\langle r\rangle = \kappa(\mu_A/2 + \mu_B + \mu_F)$ and $\langle g\rangle = \kappa(\mu_D/2 + \mu_E)$.

The expressions of autocorrelations $G_{rr}(t)$ and $G_{gg}(t)$ are obtained by trivial substitutions in the expression of $G_{gg}(t)$ from scheme I (eq. (41)), using distributions shown on Figure 2—figure supplement 4.

The red to green crosscorrelation is obtained as follows:

$$\begin{aligned}
M_{rg}(t) = \kappa \iint\cdots\int_{\mathbb{R}^5} \Bigg[& \frac{-\overline{H_3}(t-t_C) + \overline{H_3}(t-t_C-t_D)}{t_D} \\
& - \overline{H_2}(t-t_C-t_D-t_E) \\
& - \frac{\overline{H_4}(t-t_B-t_F-t_C) - \overline{H_4}(t-t_B-t_F-t_C-t_D)}{t_D\, t_A} \\
& \quad + \frac{\overline{H_4}(t-t_A-t_B-t_F-t_C) - \overline{H_4}(t-t_A-t_B-t_F-t_C-t_D)}{t_D\, t_A} \\
& + \frac{-\overline{H_3}(t-t_B-t_F-t_C-t_D-t_E) + \overline{H_3}(t-t_A-t_B-t_F-t_C-t_D-t_E)}{t_A} \Bigg] \\
& \qquad A(t_A)B(t_B)\cdots F(t_F)\ dt_A dt_B \cdots dt_F
\end{aligned} \tag{67}$$

$$= \kappa\, \overline{H_2} * \left(\overline{H} * \left(-\frac{\delta}{h_A} + \frac{A}{t}\right) * B * F - \delta\right) * C * \left(-\overline{H} * \left(-\frac{\delta}{h_D} + \frac{D}{t}\right) + D * E\right) \tag{68}$$

The green to red crosscorrelation is obtained as follows:

$$M_{gr}(t) = \kappa \iint_{\mathbb{R}^2} \left[\frac{-\overline{H_3}(t+t_C+t_D) + \overline{H_3}(t+t_C)}{t_D}\right] C(t_C)D(t_D)\ dt_C dt_D \tag{69}$$

$$= 0 \tag{70}$$

since both $\overline{H_3}$ terms are always null for $t \geq 0$.

Appendix 2D – Scheme IV

We show that, using the distributions of scheme IV (Figure 2—figure supplement 4), the correlation functions are

$$G_{rr}(t) = \frac{\kappa}{\langle r\rangle^2}\ \overline{H_2} * \left(-\overline{H_2} * \frac{A}{t^2} + \overline{H} * \left(-\frac{\delta}{h_A} + \frac{A}{t}\right) * B * D * E\right) \tag{71}$$

$$G_{gg}(t) = \frac{\kappa}{\langle g\rangle^2}\ \overline{H_2} * \left(-\overline{H_2} * \frac{D}{t^2} + \overline{H} * \left(-\frac{\delta}{h_D} + \frac{D}{t}\right) * E * F\right) \tag{72}$$

$$G_{rg}(t) = \frac{\kappa}{\langle r\rangle\langle g\rangle}\ \overline{H_2} * \left(-F + \overline{H} * \left(-\frac{\delta}{h_A} + \frac{A}{t}\right) * B * \left(-\overline{H} * \left(-\frac{\delta}{h_D} + \frac{D}{t}\right) + D * E * F\right)\right) \tag{73}$$

$$G_{gr}(t) = \frac{\kappa}{\langle r\rangle\langle g\rangle}\ \overline{H_3} * \left(-\frac{\delta}{h_D} + \frac{D}{t}\right) * E \tag{74}$$

with $\langle r\rangle = \kappa(\mu_A/2 + \mu_B + \mu_D + \mu_E)$ and $\langle g\rangle = \kappa(\mu_D/2 + \mu_E + \mu_F)$

The expressions of autocorrelations $G_{rr}(t)$ and $G_{gg}(t)$ are obtained by trivial substitutions in the expression of $G_{gg}(t)$ from scheme I (eq. (41)), using distributions shown on Figure 2—figure supplement 4.

The red to green crosscorrelation is obtained as follows:

$$\begin{aligned} M_{rg}(t) = \kappa \iint\cdots\int_{\mathbb{R}^5} \Bigg[& \frac{-\overline{H_3}(t + t_D + t_E) + \overline{H_3}(t + t_E)}{t_D} \\ & - \overline{H_2}(t - t_F) \\ & - \frac{\overline{H_4}(t - t_B) - \overline{H_4}(t - t_B - t_D) - \overline{H_4}(t - t_A - t_B) + \overline{H_4}(t - t_A - t_B - t_D)}{t_D\, t_A} \\ & + \frac{-\overline{H_3}(t - t_B - t_D - t_E - t_F) + \overline{H_3}(t - t_A - t_B - t_D - t_E - t_F)}{t_A} \Bigg] \\ & A(t_A)B(t_B)\cdots F(t_F)\ \ dt_A dt_B \cdots dt_F \end{aligned} \tag{75}$$

Remarking that the first line is always null for $t \geq 0$ and applying similar derivations as for scheme I, we find that

$$M_{rg}(t) = \kappa\, \overline{H_2} * \left(-F + \overline{H} * \left(-\frac{\delta}{h_A} + \frac{A}{t}\right) * B * \left(-\overline{H} * \left(-\frac{\delta}{h_D} + \frac{D}{t}\right) + D * E * F\right)\right) \tag{76}$$

The green to red crosscorrelation is obtained as follows:

$$M_{gr}(t) = \kappa \iint_{\mathbb{R}^2} \left[\frac{-\overline{H_3}(t - t_E) + \overline{H_3}(t - t_D - t_E)}{t_D}\right] D(t_D)E(t_E)\ \ dt_D dt_E \tag{77}$$

$$= \kappa\, \overline{H_3} * \left(-\frac{\delta}{h_D} + \frac{D}{t}\right) * E \tag{78}$$

Appendix 2E – Scheme V

We show that, using the distributions of scheme V (Figure 2—figure supplement 4), the correlation functions are

$$G_{rr}(t) = \frac{\kappa}{\langle r\rangle^2}\ \overline{H_2} * \left(-\overline{H_2} * \frac{A}{t^2} + \overline{H} * \left(-\frac{\delta}{h_A} + \frac{A}{t}\right) * B * F\right) \tag{79}$$

$$G_{gg}(t) = \frac{\kappa}{\langle g\rangle^2}\ \overline{H_2} * \left(-\overline{H_2} * \frac{D}{t^2} - (H * E)\left(H * F * \overline{C} * \frac{\overline{D}}{t}\right) - \left(H * \frac{D}{t} * E\right)(H * F * \overline{C})\right) \tag{80}$$

$$\begin{aligned} G_{rg}(t) = & \frac{\kappa}{\langle r\rangle\langle g\rangle}\ \overline{H_2} * \left(\overline{F} * C * \left(\overline{H} * \left(-\frac{\delta}{h_D} + \frac{D}{t}\right) - D * E\right)\right. \\ & \left. + \left(-\frac{\delta}{h_A} + \frac{A}{t}\right) * B * \left(-C * \overline{H_2} * \left(-\frac{\delta}{h_D} + \frac{D}{t}\right) + 1 - (H * C * D * E)(H * F)\right)\right) \end{aligned} \tag{81}$$

$$G_{gr}(t) = \frac{\kappa}{\langle r\rangle\langle g\rangle}\ \overline{H_3} * \left(\frac{\delta}{h_D} + \frac{\overline{D}}{t}\right) * \overline{C} * F \tag{82}$$

with $\langle r\rangle = \kappa(\mu_A/2 + \mu_B + \mu_F)$ and $\langle g\rangle = \kappa(\mu_D/2 + \mu_{\lceil E, F*\overline{C}*\overline{D}\rceil})$.

$\lceil X, Y\rceil$ denotes the distribution of the maximum between two numbers drawn from distributions X and Y (i.e. $H * \lceil X, Y\rceil = (H * X)(H * Y)$). Note that this, as well as eqs. (80) and (81), involve terms in H (instead of $\overline{H}$).

The expression of autocorrelation $G_{rr}(t)$is obtained by trivial substitutions in the expression of $G_{gg}(t)$ from scheme I (eq. (41)), using distributions shown on Figure 2—figure supplement 4.

The green autocorrelation is obtained as follows:

$$M_{gg}(t) = \kappa \iiiint_{\mathbb{R}^4} \left[\frac{-\overline{H_4}(t - t_D)}{t_D^2} + \frac{-\overline{H_3}(t - \max(t_E, t_F - t_C - t_D)) + \overline{H_3}(t - \max(t_E + t_D, t_F - t_C))}{t_D}\right] C(t_C)\, D(t_D)\, E(t_E)\, F(t_F)\, dt_C\, dt_D\, dt_E\, dt_F \tag{83}$$

Remarking that $\overline{H}(t) = 1 - H(t)$ and that $H(t - \max(X, Y)) = H(t - X)H(t - Y)$, we find

$$M_{gg}(t) = \kappa\, \overline{H_2} * \left(-\overline{H_2} * \frac{D}{t^2} + \iiiint_{\mathbb{R}^4} \left[\frac{H(t - t_E)H(t - t_F + t_C + t_D) - H(t - t_E - t_D)H(t - t_F + t_C)}{t_D}\right] C(t_C)\, D(t_D)\, E(t_E)\, F(t_F)\, dt_C\, dt_D\, dt_E\, dt_F\right) \tag{84}$$

$$= \kappa\, \overline{H_2} * \left(-\overline{H_2} * \frac{D}{t^2} - (H * E)\left(H * F * \overline{C} * \frac{\overline{D}}{t}\right) - \left(H * \frac{D}{t} * E\right)(H * F * \overline{C})\right) \tag{85}$$

The red to green crosscorrelation is obtained as follows:

$$\begin{aligned} M_{rg}(t) = \kappa \iint\cdots\int_{\mathbb{R}^5} \Bigg[& \frac{-\overline{H_3}(t + t_F - t_C) + \overline{H_3}(t + t_F - t_C - t_D)}{t_D} - \overline{H_2}(t - \max(t_C + t_D + t_E - t_F, 0)) \\ & - \frac{\overline{H_4}(t - t_B - t_C) - \overline{H_4}(t - t_B - t_C - t_D) - \overline{H_4}(t - t_A - t_B - t_C) + \overline{H_4}(t - t_A - t_B - t_C - t_D)}{t_D\, t_A} \\ & + \frac{-\overline{H_3}(t - t_B - \max(t_C + t_D + t_E, t_F)) + \overline{H_3}(t - t_A - t_B - \max(t_C + t_D + t_E, t_F))}{t_A} \Bigg] \\ & A(t_A)\, B(t_B) \cdots F(t_F)\, dt_A\, dt_B \cdots dt_F \end{aligned} \tag{86}$$

Noting that, over $t \geq 0$, a term of the form $\overline{H_n}(t - \max(a, b))$ simplifies to $\overline{H_n}(t - a)$ if $b \leq 0$, and applying the same derivation principle as for $M_{gg}(t)$, we find that

$$\begin{aligned}
M_{rg}(t) = \kappa\, \overline{H_2} * \Bigg(& \overline{F} * C * \overline{H} * \left(-\frac{\delta}{h_D} + \frac{D}{t}\right) - \overline{F} * C * D * E \\
& - \overline{H} * \left(-\frac{\delta}{h_A} + \frac{A}{t}\right) * B * C * \overline{H} * \left(-\frac{\delta}{h_D} + \frac{D}{t}\right) \\
& + \left(-\frac{\delta}{h_A} + \frac{A}{t}\right) * B * \iiiint_{\mathbb{R}^4} \Big[1 - H(t - t_C - t_D - t_E) H(t - t_F))\Big] \\
& \qquad C(t_D)\, D(t_D)\, E(t_E)\, F(t_F)\; dt_C\, dt_D\, dt_E\, dt_F \Bigg) \qquad (87)
\end{aligned}$$

$$\begin{aligned}
= \kappa\, \overline{H_2} * \Bigg(& \overline{F} * C * \left(\overline{H} * \left(-\frac{\delta}{h_D} + \frac{D}{t}\right) - D * E\right) \\
& + \left(-\frac{\delta}{h_A} + \frac{A}{t}\right) * B * \left(-C * \overline{H_2} * \left(-\frac{\delta}{h_D} + \frac{D}{t}\right) + 1 - (H * C * D * E)(H * F)\right)\Bigg) \qquad (88)
\end{aligned}$$

The green to red crosscorrelation is obtained as follows:

$$M_{gr}(t) = \kappa \iiint_{\mathbb{R}^3} \left[\frac{-\overline{H_3}(t + t_C + t_D - t_F) + \overline{H_3}(t + t_C - t_F)}{t_D}\right] C(t_C)\, D(t_D)\, F(t_F)\; dt_C\, dt_D\, dt_F \qquad (89)$$

$$= \kappa\, \overline{H_3} * \left(\frac{\delta}{h_D} + \frac{\overline{D}}{t}\right) * \overline{C} * F \qquad (90)$$

Appendix 3 Bayesian Information Criterion (BIC)

To do model comparison, we used the Bayesian Information Criterion (BIC) [3]

$$\text{BIC} = \chi^2 + k \log N \qquad (91)$$

where, χ^2 is directly obtained from the non-linear least-square fit, k is the number of parameter of the model and N the number of data points used in the fit. The BIC penalizes models with more parameters so that if two models fit equally well the data, the simplest one will be retained. The smallest the BIC, the better the model.

Supplementary references

[1] A. Papoulis, "Probability, random variables, stochastic processes," Jan. 1991.

[2] W. C. Van Etten, "Introduction to Random Signals and Noise," pp. 1–271, Jan. 2006.

[3] S. Konishi and G. Kitagawa, Information Criteria and Statistical Modeling. Springer, 2008.
